# Exploration of the Dietary and Lifestyle Behaviors and Weight Status and Their Self-Perceptions among Health Sciences University Students in North Lebanon

**DOI:** 10.1155/2016/9762396

**Published:** 2016-06-27

**Authors:** Germine El-Kassas, Fouad Ziade

**Affiliations:** ^1^Nutrition & Dietetics Department, Faculty of Health Sciences, Beirut Arab University, Triopoli 1301, Lebanon; ^2^Faculty of Public Health, Lebanese University, Tripoli 1300, Lebanon

## Abstract

University students may experience significant environmental changes that exert a negative influence on the quality of their diet and lifestyle. There is scarcity of data concerning the dietary and lifestyle behaviors and weight status of students in the health field in North Lebanon. To investigate these data, a cross-sectional survey was conducted including 369 health sciences students aged 18–25 chosen from four public and private universities in North Lebanon. Data were collected using a standardized interview questionnaire to determine sociodemographic, dietary, and lifestyle behaviors, appetite changes, stress related dietary behaviors, and food cravings, as well as self-perceptions of dietary adequacy, physical activity levels, and weight status. Body mass index was assessed. Results had revealed significant differences in some of the dietary consumption patterns and weight status among seniors compared to juniors. However, the overall prevalence of overweight and obesity recorded 32.2% and the dietary consumption patterns fall below recommended levels. Multivariate regression analysis showed that parental obesity, comfort eating, increased appetite, food cravings, and stressful eating were associated with increased risk of obesity while a healthy diet score was associated with decreased risk. The study's findings call for tailoring culture specific intervention programs which enable students to improve their dietary and lifestyle behaviors and control stress.

## 1. Introduction

Healthy eating habits and lifestyle play a key role in the prevention of chronic noncommunicable diseases (NCDs) such as diabetes, cardiovascular diseases, cancer, Alzheimer's disease, and hepatic steatosis [1–3]. While prevention of nutrition-related noncommunicable diseases has become a worldwide challenge [4], it has been documented that NCDs share four main behavioral risk factors all of which will likely escalate in developing countries including insufficient physical activity and unhealthy diet/obesity and tobacco use [5]. Alarmingly, NCD-related mortality is occurring at earlier ages in developing countries [6]. Lebanon as well as other countries in the Middle East and North Africa region has faced an epidemiologic transition over the past decades, with the result being marked changes in food consumption patterns and lifestyle behaviors [7]. The traditional Mediterranean healthy food habits have been replaced by more westernized food habits, which are characterized by low intake of dietary fiber, vegetables, and fruit and high intake of foods rich in fat, sugar, and salt [7]. Subsequently, an alarming increase in nutrition-related chronic diseases has been reported by national and community-based surveys which could be considered as an important public health problem in Lebanon [8, 9].

The human choice of foods is described as a complex process involving a multiplicity of influencing aspects such as the socioeconomic and cultural level and availability of food, as well as the educational level and age range of a person [10]. University student populations are widely reported to engage in high rates of physical inactivity, sedentary behaviors, and unhealthy dietary behaviors including skipping meals, inadequate snacking, high consumption of fast foods, and insufficient consumption of fruit and vegetables [11–15]. Insufficient physical activity and unhealthy diet can lead to an array of negative physical changes in the youth such as high blood pressure and overweight/obesity, which can trigger NCDs in adulthood [4]. NCDs risk factors can be less damaging if addressed early in life, when habits are not yet well established [16]. Minimizing risk factors for NCDs, particularly during early adulthood, offers the opportunity for better health, more years of productivity, and lower health care costs [4]. A focus on strengthening protective factors and earlier investment in prevention of NCDs among young people and particularly university students is therefore essential. In this respect, it was stated that, in the age range of 18 to 24 years of many university students, the establishment of healthy life behaviors, including eating behavior, may have a lasting impact on the health of these individuals and consequently on the health of their future families [17]. This becomes even more relevant regarding university students in the health area, who will perpetuate their acquired habits not only for themselves and their relatives, but also for the future community of patients covered by their professional activity [18].

It is often assumed that students in the health field had greater knowledge than other students, yet there is no evidence to indicate that this knowledge is translated into healthy dietary and lifestyle practices [18]. Some studies among university students suggested that students in the health field may have more favorable food choices which can be reflected on attaining a healthier nutritional status than other university students [18, 19]. On the contrary, data reported by other studies reported high prevalence of unhealthy dietary choices and lack of physical activity [20, 21].

On the basis of these premises, it is necessary to observe the impact of the university context on the lifestyle and eating behavior of the students in the health field in order to identify factors that influence their nutritional status, dietary and lifestyle habits, and quality of life throughout the years of study to enable tailoring of the most relevant health-promoting interventions. Limited studies had explored the dietary and lifestyle behaviors of university students in Lebanon but none as far as is known had thoroughly investigated the role of the field of study and its associations with dietary and lifestyle habits and weight status evolution. Therefore, this study was conducted to explore the dietary habits, physical activity, sedentary behaviors, and weight status as well as self-perception of changes in appetite, body weight, diet quality, and physical activity levels through a representative sample of junior and senior health sciences students in both public and private universities in North Lebanon.

## 2. Methods 

### 2.1. Study Design and Participants

Through a cross-sectional study design, a survey was conducted in Tripoli during the period between September and December 2015. The study participants were students aged 18–25 years studying a major in the health field either in their first semester of study (junior level) or in the fifth semester and above (senior level) in the selected universities.

### 2.2. Sampling Procedure

Based on previously reported data [22], a sample size of 170 junior students and 170 senior students was needed to detect a difference of 5% in the body mass index between the two groups with power equal to 80% and significance level (alpha) equal to 5%. All universities in North Lebanon which has a faculty for health sciences were briefed through the deans of these faculties, and four out of six universities agreed to participate in the study, namely, the Lebanese University (the only public university in North Lebanon), Beirut Arab University (BAU), Tripoli Campus, Al Jinan University, and Al Manar University (MUT), during the fall semester 2015/2016. The inclusion criteria were being a regular student either in the first semester of study (junior level) or in the fifth semester and above (senior level) within the age group of 18–25 years. The exclusion criteria were any student having any physical motor disability or having any chronic metabolic disease like diabetes mellitus or chronic kidney or liver diseases and those on regular intake of specific drugs that may affect appetite or weight control. Preliminary information was provided about the purpose, the protocol, and the method of the study, including the guarantee of anonymity. None of the students refused to participate and all students fulfilling the inclusion criteria were recruited; only those who were absent on the days of data collection were excluded. All subjects gave their informed consent for inclusion before they participated in the study. The study was conducted in accordance with the Declaration of Helsinki, and the protocol was approved by the Ethics Committee of BAU.

### 2.3. Data Collection

A structured anonymous interview questionnaire was developed by the author based on previously published instruments which has been standardized and validated to be used among university students [22–24]. During break time, the interview questionnaires were applied in the classrooms, by trained researchers (who had participated in previous training to standardize the data collection procedures), and under continuous supervision of a professor. The questionnaire included questions to assess the sociodemographic characteristics, appetite changes, weight and physical activity perception and changes, dietary and food intake patterns, and physical activity and lifestyle behaviors followed by anthropometric measurements.

### 2.4. Measures

#### 2.4.1. General and Sociodemographic Characteristics

Questions inquiring about age, gender, type of university, type of major of study, number of semesters since joining university, the type of current residence and living conditions either alone or with family or friends, field of study, educational level of both parents, and parental obesity (of one or both parents) were asked to define the general and sociodemographic characteristics of the study sample.

#### 2.4.2. Anthropometric Measurements

Anthropometric measurements including weight, height, and waist circumference were assessed by trained researchers using standardized techniques [25] and calibrated scales. Standing height was measured to the nearest 0.1 cm without shoes, using a stadiometer. Participants wearing light clothes were weighed to the nearest 0.1 kg, on an electronic scale which was first calibrated using a standard weight and rechecked daily [26]. Body mass index (BMI) was calculated using the formula body weight (Kg)/height (m^2^) in accordance with the World Health Organization (WHO) criteria for overweight and obesity classification [25]. BMI values were classified into four categories: underweight (BMI ≤ 18.5 kg/m^2^), normal weight (BMI between 18.5 and 24.9 kg/m^2^), overweight (BMI between 25 and 29.9 kg/m^2^), and obese (≥30 kg/m^2^) [27].

#### 2.4.3. Dietary Intake Assessment

The dietary and food intake patterns including the regularity of meal consumption, regular breakfast intake, numbers of meals, and number of snacks were assessed. A semiquantitative food frequency questionnaire (FFQ) was used covering different food categories (including the five basic food categories typically consumed by the Lebanese population). The FFQ used in this study was adapted from the questionnaire earlier administered in the Lebanese population and other studies conducted among students living in the Mediterranean region and Arab countries [28–30]. The items used were fruits and fresh fruit juices, vegetables (raw and cooked), milk and dairy products, legumes, oils (olive, corn, and canola sunflower), carbonated beverages, fruit juices, sweet snacks (cakes, chocolates), salty snacks (chips), fast foods (burgers and pizza), and fried foods. Intake categories included the number of servings per day and per week as follows: 6/day, 4-5/day, 2-3/day, one/day, 5-6/w, 2–4/w, once/w, 1–3/m, and never.

According to the method established by Papadaki and Scott [30], the frequency of consumption of each food and beverage category was transformed as follows: the frequency value “never” was transformed to “0 times per week,” “1–3 servings per month” was transformed to “0.5 servings per week,” “once per week” was transformed to “1 serving per week,” “2–4 servings per week” became “3 servings per week,” “5-6 servings per week” became “5.5 servings per week,” “once per day” became “7 servings per week,” “2-3 servings per day” became “17.5 servings per week,” “4-5 servings per day” became “31.5 servings per week,” and “6 servings per day” was transformed to “48 servings per week.” The mean intake/week of each food item was then calculated.

A diet score was also developed based on the food frequency data to assess the dietary adequacy of the students. For this purpose, intake categories were scored increasingly from 1 to 6 for healthy food items including fruits, vegetables, fruit juices, raw and cooked vegetables, legumes, healthy oils, milk, and dairy products. Inverse coding was assigned for unhealthy food items including carbonated beverages, sweet snacks (chocolate and cake), butter or ghee, pastries, pizza, burgers, and fried food. The total score was derived by summing the scores for all the food items included in the questionnaire. The total score varied from 17, the least healthy, to 112, the healthiest diet score.

#### 2.4.4. Physical Activity and Lifestyle Variables

The short form of the International Physical Activity Questionnaire (IPAQ) for the last 7 days (IPAQ-S7S) [31] was used in order to assess the physical activity level of the students. We followed the instructions given in the IPAQ manual for reliability and validity. The IPAQ short form asks about three specific types of activity undertaken in leisure time, work-related and transport-related activity and domestic activities. The specific types of activity that were assessed are walking, moderate-intensity activities, and vigorous-intensity activities; frequency (measured in days per week) and duration (time per day) are collected separately for each specific type of activity. The items were structured to provide separate scores on walking, moderate-intensity activity, and vigorous-intensity activity as well as a combined total score to describe overall level of activity. Computation of the total score requires summation of the duration (in minutes) and frequency (days) of walking, moderate-intensity activity, and vigorous-intensity activity. We categorized physical activity (short form) according to the official IPAQ scoring protocol [32] as low, moderate, and high.

### 2.5. Data Analysis

Frequencies, means, and standard errors (SE) were used to describe various sociodemographic and lifestyle behaviors and dietary and anthropometric characteristics. Chi square test and Student's *t*-test were used to compare proportion and means, respectively. The Chi square for trend in the comparison of ordinal independent variables was also applied. The odds of being overweight or obese were determined using multivariate binary logistic regression analysis models where all the covariates were entered simultaneously each as an independent variable. All the analysis was two-tailed and a *p* value of <0.05 was considered statistically significant. All the analysis was performed using the Statistical Package for the Social Sciences (version 21, Armonk, NY, USA).

## 3. Results

### 3.1. Characteristics of the Subjects

Table 1 describes students' sociodemographic characteristics grouped by BMI categories. It shows that a total of 369 health sciences students with a mean age of 19.60 ± 1.67 years were included in the analysis; 86.4% of them were females and 13.6% were males. A statistically significant difference in the weight status (*p* = 0.042) among seniors compared to juniors was demonstrated as lower percentages of underweight status (6.7% versus 1.7%), overweight status (29% versus 26.1%), and obese status (5.7% versus 3.4%) among seniors and juniors, respectively. No significant differences were detected regarding the type of university, current residence, and living conditions. The educational levels of the mother and father were reaggregated as preparatory and below and senior and above (*p* = 0.445, *p* = 0.508). In addition, the nutrition major was compared against other types of majors but no statistically significant difference was detected (*p* = 0.648). Chi square test was repeated and the reported *p* value represents results after aggregating the responses in order to avoid very low cells.

### 3.2. Appetite, Lifestyle, Physical Activity, and Dietary Behaviors

The appetite status, dietary intake patterns, and sedentary behaviors were compared by the level of study as shown in Table 2. Overall, a tendency towards a healthier lifestyle and dietary behaviors was detected among the senior group compared to juniors. There were statistically significant differences as regards current appetite compared to before going to university, preferred activities during free time, and eating meals while watching TV or computer (*p* = 0.041, *p* = 0.047,  and  *p* = 0.002, resp.). In addition, seniors tended to have healthier preferences concerning types of drinks between meals, meal preparation methods, consumption of coffee or tea directly after meals, and lower frequency in stress induced eating; however, none of these had reached a statistically significant level as shown in Table 2. On the other hand, seniors compared to juniors reported lower frequency of regular breakfast intake and number of main meals and snacks, with no statistically significant differences (see Table 2).

### 3.3. Agreement between Perceived and Actual Weight Status, Diet Quality, and Physical Activity Level

Table 3 shows that, overall, 63.4% of the sample were in the normal BMI category, while 32.2% were considered overweight and obese and only 4.2% fall into the underweight category with a statistically significant difference between seniors and juniors (*p* = 0.042). About two-thirds (66.9%) and more than half (55.3%) of the studied sample correctly perceived their weight status and diet quality, respectively, with no statistically significant difference between seniors and juniors. Concerning physical activity, the table shows that 41.2% of the studied sample had been evaluated as having low physical activity levels with no statistically significant difference between the senior and junior groups. There was a statistically significant difference between seniors and juniors (*p* < 0.001) as regards current physical activity levels compared to preuniversity life. A statistically higher proportion of seniors had shown agreement of perception of physical activity level where 59.1% of the seniors compared to only 48.7% of the juniors had correctly perceived their physical activity levels (*p* = 0.046).

### 3.4. Dietary Consumptions Patterns

Based on the level of study, analysis of the semiquantitative FFQ had shown some significant differences between junior and senior health sciences university students with respect to their consumption of individual food categories regularly consumed by the Lebanese population. Seniors consumed statistically significant higher mean weekly serving of some healthy food items as olive oil and low fat yoghurt (*p* = 0.035  and  *p* = 0.024, resp.). In contrast, juniors had statistically significant higher mean weekly servings consumption of croissants and pizza (*p* = 0.001). In addition, higher mean weekly serving intake was reported by juniors compared to seniors concerning high fat foods like burger, cakes, and fried potatoes but the difference did not reach a significant level as shown in Table 4.

### 3.5. Association between Overweight/Obesity and Sociodemographic, Dietary, and Lifestyle Behaviors among University Students

Multivariate binary logistic regression analysis revealed that parental obesity (OR: 0.468, 95% CI: 0.274, 0.802), food cravings for high sugary or high fat foods (OR: 3.054, 95% CI: 1.282, 7.272), increased appetite compared to before entering university (OR: 0.466, 95% CI: 0.255, 0.851), stress induced eating (OR: 2.672, 95% CI: 1.198, 5.960), and comfort eating (OR: 0.581, 95% CI: 0.343, 0.984) were associated with statistically significant higher odds for being overweight and obesity. On the other hand, the total healthy food score was associated with statistically significant lower odds for the development of obesity (OR: 1.055, 95% CI: 1.013, 1.099) (Table 5).

## 4. Discussion

The findings of the present study identified some significant differences between health sciences students in the senior level compared to juniors presented as an overall healthier weight status, few better dietary habits, and an improved perception of the physical activity level. In addition, the present findings detected some significant associations with the development of overweight/obesity among the studied sample.

A number of studies had investigated the determinants of eating behavior and food choices among university students. Most of these studies concluded that several factors influence the dietary choices and behaviors including individual factors (e.g., taste preferences, self-discipline, state of mind or stress, body image, time and convenience, dietary knowledge, past eating habits, physical activity level, and daily rhythm), physical environment (e.g., availability and accessibility, appeal and prices of food products), and university characteristics, such as university lifestyle and exams [10, 33]. Studies concerning the dietary and lifestyle behaviors of students in the health careers reported controversial results but most of these studies indicated that the majority of these students have a high tendency to engage in unhealthy dietary and lifestyle habits including meal skipping, low fruits and vegetables intake, high fast food consumption, and minimal physical activity [18, 19, 34]. Analysis of the dietary habits of the students in the present sample revealed adoption of several undesired dietary habits. Our results had shown that about half of the students (both seniors and juniors) have less than three meals per day and unhealthy snacking patterns. In addition, more than one-third of the students in the present sample (38.5%) skip breakfast and most of them prefer unhealthy food choices for breakfast. This was consistent with recent findings among nursing university students from Greece [35] but the percentage of breakfast skippers in the present study was lower than the reported data among Bahraini health sciences students (56%) [21]. It is also worth noting that the present breakfast intake data could be considered better than data reported among non-health sciences Lebanese university students living in Tripoli or Beirut [12, 22]. It is well documented that high consumption of fruits and vegetables is associated with a lower risk of chronic diseases related mortality especially cardiovascular diseases and cancer [36]. However, the mean weekly intake for fruits and cooked and raw vegetables was less than the recommended level for both seniors and juniors in the present study. The current findings were in agreement with previously reported data among students in the health careers [21, 34].

The dietary behavior of eating while watching TV had been recently studied as a risk factor of alteration of the energy balance and increasing caloric intake. Some studies have correlated it to obesity [37] while others indicated that the impact of TV watching on the amount of food consumed is dependent on how much the viewer is being interested and engaged in the attended program [38, 39]. Junior students among the current studied sample had reported statistically significant higher intake of meals while watching TV than seniors. Given that the food choices of a large proportion of juniors are not healthy, the behavior of having meals while watching TV may pose a risk for the development of obesity later and should be targeted by an appropriate behavioral modification program.

Body weight and its perception are important aspects of health and constitute a significant role in physical and mental well-being [40]. Regardless of whether a person is underweight, normal, or overweight, weight perception is an important determinant of nutritional habits and weight management [41, 42]. Analysis of the present data estimated a relatively high rate of discrepancy between perceived and actual weight status as one-third of the studied sample misclassified their weight status with no statistically significant difference between juniors and seniors. This was typically in harmony with previously reported data among female university students living in Karachi [42]. The current unexpected finding that senior students misperceived their weight status more frequently compared to juniors was related to seniors who underestimated their weight status as being underweight though they were in the normal weight category. This could be related to the cultural based distorted body image prevailing in the Arab countries and in the Middle East which alters appropriate self-perception of body weight at a young age [43, 44].

Regarding the actual weight status, our findings indicated significant difference in the weight status which had been reflected as higher prevalence of normal weight and lower prevalence of underweight, overweight, and obese students in the senior group compared to juniors, respectively. This finding was in agreement with reported data by nursing students in Spain who showed lower prevalence of overweight and obesity among fourth-year (16%) compared to first-year (22.1%) students [45]. In contrast, 48.8% of the students in the health careers in Brazil reported gaining weight after entering the university [46]. The healthier weight status identified in the current study may be attributed to the detected tendency towards a healthier eating pattern and food choices among seniors compared to juniors regarding frequency of consumption of some food items which was demonstrated as significantly higher mean weekly consumption of olive oil and low fat yoghurt. In addition, significantly lower consumption of high fat foods like pizza and croissants was also identified among seniors compared to juniors in the present sample. This lower consumption of high fat foods may contribute to the improvement in the weight status and this was further indicated by the finding that a more healthy food score is associated with a lower risk for the development of obesity. Alternatively, this healthier eating pattern and better food choices could also explain why seniors had lower prevalence of overweight/obese status in spite of the reported statistically significant increased appetite which if not coupled with healthy food choices will lead to excessive caloric intake, resulting in overweight and obesity.

Unexpectedly, no statistically significant difference between students majoring in nutrition and students studying other majors in the health fields was detected. In contrast to our findings, the lowest percentage of students with weight problems corresponded to the majors in nutrition, in which no young people with overweight problems were identified and only 4% were obese among health sciences students in Mexico [47]. This could suggest that factors other than attaining better nutritional knowledge could have stronger influence on dietary choices and lifestyle behaviors of the present sample which predominantly shape their weight status or there may be some barriers to translate knowledge into practice as had been previously suggested [18]. It is also noteworthy that, considering the mean age of the studied sample (19.60 ± 1.67) and assuming that health sciences student are more knowledgeable about the risks of obesity, the figures of the overall prevalence of overweight and obesity are worrisome especially if compared to previously reported lower prevalence rates among university students in Lebanon [12, 22]. This should trigger a thorough investigation of the factors associated with obesity among health sciences students.

A mounting body of evidence suggests that obesity is a complex multifactorial problem implying genetic, dietary, environmental, and behavioral factors [48]. In agreement, analysis of the factors associated with obesity in the present study had shown a diversity of factors including hereditary, dietary, and emotional eating like increased eating in relation to stress or stressful eating, food craving, and eating for comfort. Parental obesity, which may be related to either genetic or the family home environment, had been previously documented as one of the predictors of obesity in young adults [49, 50]. In accordance, parental obesity was found to be significantly associated with increased risk for the development of obesity among health sciences students of the current study. In addition, increased appetite (compared to preuniversity life) among students in the present sample was detected as a significant risk factor for obesity development. On combining the enhanced appetite and the finding that the majority of students have unhealthy dietary choices (low consumption of fruits and vegetables and high intake of high fat foods) which was indicated by their mean weekly consumption of different food items and the mean total diet score, we can deduce that enhanced appetite among the present sample may result in obesity. On the other hand, a healthy total diet score was associated with decreased risk for obesity development. It has been documented in the literature that regular breakfast intake is associated with lower body weight [12, 51]. However, regular breakfast intake in the present study was not a significant protective factor against the development of obesity. This could be explained by the finding that the majority of the students in the present sample preferred calorie dense/high fat choices (Mankoush or pastries) for their breakfast meals.

Researchers have suggested a positive association between weight gain and psychological stresses related to university life [52, 53]. Stress may contribute to changes in dietary behaviors that lead to weight change. Moreover, stress seems to be associated with a greater preference for energy- and nutrient-dense foods, namely, those that are high in sugar and fat [54]. Several studies among university students reported a positive association between perceived stress and weight gain [55, 56]. These findings were in accordance with our results which had shown that several eating behaviors related to stress such as eating for comfort, stress induced eating, and cravings for high fat/high sugar foods were associated with increased risk for the development of obesity.

One of the challenges that university students have to face while transitioning into university life is deciding to engage in either physical activity or sedentary behaviors. Research data have suggested two different theories regarding the relationship between sedentary behaviors and physical activity; they are either negatively correlated or uncorrelated [57–59]. Our findings confirmed the latter theory, as there was high prevalence of sedentary behaviors (60% of the studied sample reported spending more than two hours daily watching TV or using a computer); meanwhile, evaluation of the physical activity levels (MET values) showed that 60% of the studied sample are categorized as having moderate/high physical activity levels. These physical activity levels could be considered more favorable than previously reported data among university students in Arab or developing countries and in Lebanon [11, 12, 21, 60]. Although a statistically significant higher proportion of the senior group in the current sample had reported that their physical activity has increased compared to before joining university, however, seniors became significantly less engaged in sport activities and they preferred only walking. These two findings combined could be attributed to the fact that students in the senior level in the health sciences field mostly become more engaged in activities related to their studies like hospital training and research projects which could limit the spare time available for sports practice; meanwhile, they perceive that they became more active. In addition, it has been previously suggested that students choose their activities according to the available time and convenience [61]. This could offer an additional explanation to why seniors are less engaged in sports activities. The current data had also shown a significantly better ability of seniors to perceive their physical activity levels more appropriately. This may be related to the fact that seniors are more knowledgeable than juniors [18] and so could better evaluate their physical activity levels.

Although the link between enhanced physical activity and lowering the risk of obesity among university students has been established in a number of previous studies [62, 63], the present study, however, did not show a significant association between physical activity and overweight and obesity. Similar findings had been reported by other researchers who did not find a link between physical inactivity and overweight/obesity either for male or for female university students despite showing that the men are more likely to engage in physical exercise in their free time [64]. Other studies indicate that the relationship between BMI and physical activity occurs only among men [61, 65].

## 5. Conclusion 

The present study results had pinpointed some association between the level of health sciences studies and some of the dietary habits, physical activity, and sedentary behaviors as well as weight status and their perceptions among Lebanese students in the health fields. Nevertheless, the present data had shown relatively alarming prevalence of overweight/obesity, unhealthy dietary practices, and lifestyle behaviors that should be targeted and modified. Taken together, these findings call for the elaboration of university based health-promoting multisectorial integrated programs. These programs may serve as a sustainable way to support healthful lifestyles for these university students. Based on the results of this study, specific behavioral intervention programs should be implemented to ensure the opportunity to overcome barriers to adopt healthy dietary and lifestyle behaviors. Implementation of such tailored programs could ensure optimal long-term health of future health care professionals who will serve as positive patient role models. The present study may also serve as baseline data for comprehensive longitudinal studies which could identify ways to improve the dietary patterns and lifestyles in the whole university population.

## Figures and Tables

**Table 1:** Sociodemographic characteristics grouped by BMI categories.

	Total (*n* = 369)		Underweight (*n* = 16)		Normal (*n* = 234)		Overweight (*n* = 102)		Obese (*n* = 17)		Test of sig.	*p*
	Number	%	Number	%	Number	%	Number	%	Number	%		
*Age*												
Min.–max.	17.0–25.0		17.0–22.0		17.0–25.0		17.0–25.0		17.0–21.0		*F* = 1.874	0.134
Mean ± SD	19.60 ± 1.67/sqrt (369)		18.94 ± 1.34/4		19.61 ± 1.63		19.78 ± 1.84		19.06 ± 1.09			

*Gender*												
Male	50	13.6	2	4.0	27	54.0	17	34.0	4	8.0	*χ* ^2^ = 3.418	^MC^ *p* = 0.310
Female	319	86.4	14	4.4	207	64.9	85	26.6	13	4.1		

*University*												
Public	209	56.6	6	2.9	139	66.5	57	27.3	7	3.3	*χ* ^2^ = 4.792	0.188
Private	160	43.4	10	6.3	95	59.3	45	28.1	10	6.3		

*Level*												
Junior	193	52.3	13	6.7	113	58.6	56	29.0	11	5.7	*χ* ^2^ = 8.209^*∗*^	0.042^*∗*^
Senior	176	47.7	3	1.7	121	68.8	46	26.1	6	3.4		

*Living conditions*												
With family	341	92.4	15	4.4	214	62.8	97	28.4	15	4.4	*χ* ^2^ = 2.090	^MC^ *p* = 0.492
Away from home	28	7.6	1	3.6	20	71.4	5	17.9	2	7.1		

*Residence area*												
Urban	250	67.8	12	4.8	155	62.0	71	28.4	12	4.8	*χ* ^2^ = 0.853	0.837
Rural	119	32.2	4	3.4	79	66.3	31	26.1	5	4.2		

*Educational level of father*												
Illiterate	28	7.6	0	0.0	18	64.3	10	35.7	0	0.0	*χ* ^2^ = 13.042	^MC^ *p* = 0.571 (0.445)^★★^
Primary school	82	22.3	3	3.7	51	62.2	26	31.7	2	2.4		
Prep. school	75	20.4	3	4.0	44	58.7	21	28.0	7	9.3		
Secondary school	58	15.8	3	5.2	40	69.0	12	20.8	3	5.2		
Above secondary school	53	14.4	4	7.5	29	54.7	18	34.0	2	3.8		
University	72	19.6	3	4.2	51	70.8	15	20.8	3	4.2		

*Educational level of mother*												
Illiterate	16	4.3	0	0.0	7	43.8	8	50.0	1	6.3	*χ* ^2^ = 14.984	^MC^ *p* = 0.394 (0.508)^★★^
Primary school	64	17.4	2	3.1	42	65.6	20	31.3	0	0.0		
Prep. school	64	17.4	2	3.1	41	64.1	16	25.0	5	7.8		
Secondary school	76	20.7	3	3.9	49	64.6	22	28.9	2	2.6		
Above secondary school	68	18.5	3	4.4	47	69.1	15	22.1	3	4.4		
University	80	21.7	6	7.5	48	60.0	20	25.0	6	7.5		

*Major of the participant*												
Health and environment	16	4.3	0	0.0	13	81.2	3	18.8	0	0.0	*χ* ^2^ = 11.524	0.486 (0.684)^★★^
Medical lab	86	23.3	5	5.8	51	59.3	25	29.1	5	5.8		
Medical social assistance	20	5.4	0	0.0	12	60.0	8	40.0	0	0.0		
Nursing	124	33.6	7	5.6	75	60.6	35	28.2	7	5.6		
Nutrition	89	24.1	4	4.5	60	67.4	22	24.7	3	3.4		
Physiotherapy	13	3.5	0	0.0	6	46.2	6	46.2	1	7.6		
Radiology	21	5.7	0	0.0	17	81.0	3	14.2	1	4.8		

^*∗*^Statistically significant at *p* ≤ 0.05.

^★★^Chi square test was repeated and the reported *p* value represents results after aggregating the responses in order to avoid very low cells.

MC: Monte Carlo for Chi square test.

**Table 2:** Appetite changes and dietary and lifestyle behaviors.

		Total (*n* = 369)		Junior (*n* = 193)		Senior (*n* = 176)		*χ* ^2^	*p*
		Number	%	Number	%	Number	%		
Current appetite status compared to before going to university	Same	126	34.1	77	39.9	49	27.8	6.405^*∗*^	0.041^*∗*^
	Increased	127	34.4	58	30.1	69	39.2		
	Decreased	116	31.4	58	30.1	58	33.0		

Eating in response to exam related stress	No	152	41.2	71	36.8	81	46.0	4.135	0.242
	Yes	167	45.3	91	47.2	76	43.2		
	Sometimes	44	11.9	27	14.0	17	9.7		
	Rarely	6	1.6	4	2.1	2	1.1		

Number of main meals	One	16	4.3	4	2.1	12	6.8	5.934	0.115
	Two	169	45.8	86	44.6	83	47.2		
	Three	162	43.9	91	47.2	71	40.3		
	More than three	22	6.0	12	6.2	10	5.7		

Number of snacks	Zero	21	5.7	9	4.7	12	6.8	2.580	0.631
	One	111	30.1	62	32.1	49	27.8		
	Two	133	36.0	68	35.2	65	36.9		
	Three	63	17.1	30	15.5	33	18.8		
	More than three	41	11.1	24	12.4	17	9.7		

Types of snacks	No snacks	4	1.1	0	0.0	4	2.3	2.036	^MC^ *p* = 0.156
	Fruit/fruit juice/yogurt	85	23.0	50	25.9	35	19.9		
	Biscuits/cakes	16	4.3	12	6.2	4	2.3		
	Fried potatoes	6	1.6	3	1.6	3	1.7		
	Sweets/chocolate/cake	70	19.0	38	19.7	32	18.2		
	Combinations	181	49.1	86	44.6	95	54.0		
	Other	7	1.9	4	2.1	3	1.7		

Regular breakfast intake	Yes	227	61.5	125	64.8	102	58.0	1.805	0.179
	No	142	38.5	68	35.2	74	42.0		

Type of preferred breakfast	Mankoush or pastries	329	89.2	179	92.7	150	85.2	7.605	0.099
	Biscuits	7	1.9	1	0.5	6	3.4		
	Cereal	8	2.2	3	1.6	5	2.8		
	Sandwich	13	3.5	4	2.1	9	5.1		
	Other	12	3.3	6	3.1	6	3.4		

Preferred method for food preparation for main meals	Frying	127	34.4	70	36.3	57	32.4	0.831	0.660
	Boiling	91	24.7	48	24.9	43	24.4		
	Grilling	151	40.9	75	38.9	76	43.2		

Consumption of energy drinks	Never	283	76.7	141	73.1	142	80.7	6.579	0.142
	Rarely	34	9.2	19	9.8	15	8.5		
	1-2 times per week	42	11.4	24	12.4	18	10.2		
	2-3 times per week	7	1.9	6	3.1	1	0.6		
	More than 3 times/week	3	0.8	3	1.6	0	0.0		

Eating for comfort	Yes	209	56.6	106	54.9	103	58.5	0.486	0.486
	No	160	43.4	87	45.1	73	41.5		

Intake of coffee or tea directly after meals	Yes	145	39.3	82	42.5	63	35.8	1.728	0.189
	No	224	60.7	111	57.5	113	64.2		

Cravings for high fat/sugar foods	Never	250	67.8	129	66.8	121	68.8	1.332	0.856
	Once/month	38	10.3	23	11.9	15	8.5		
	2–4 times	32	8.7	17	8.8	15	8.5		
	2-3 times	28	7.6	14	7.3	14	8.0		
	4 times	21	5.7	10	5.2	11	6.3		

Preferred activities during free time	Walking	38	10.3	14	7.3	24	13.6	9.639^*∗*^	0.047^*∗*^
	Watching TV/listening to music	250	67.8	127	65.8	123	69.9		
	Practicing a sport	49	13.3	30	15.5	19	10.8		
	Shopping	22	6.0	14	7.3	8	4.5		
	Other	10	2.7	8	4.1	2	1.1		

Screen time	1 h-2 h a day	149	40.4	83	43.0	66	37.5	1.611	0.657
	3 h-4 h a day	136	36.9	70	36.3	66	37.5		
	5 h-6 h a day	48	13.0	22	11.4	26	14.8		
	More than 6 h a day	36	9.8	18	9.3	18	10.2		

Eating meals while watching TV or at the computer	Yes	219	59.3	129	66.8	90	51.1	9.409^*∗*^	0.002^*∗*^
	No	150	40.7	64	33.2	86	48.9		

Smoking status	Never	284	77.0	152	78.8	132	75.0	4.074	0.254
	Previous smoker	3	0.8	0	0.0	3	1.7		
	Occasionally	43	11.7	20	10.4	23	13.1		
	Regular smoker	39	10.6	21	10.9	18	10.2		

^*∗*^Statistically significant at *p* ≤ 0.05.

MC: Monte Carlo for Chi square test.

**Table 3:** Agreement between perceived and measured weight status, physical activity, and diet quality.

	Total (*n* = 369)		Junior (*n* = 193)		Senior (*n* = 176)		*χ* ^2^	*p*
	Number	%	Number	%	Number	%		
*Perceived current body weight*								
Underweight	44	11.9	20	10.4	24	13.6	2.312	0.518 (0.130)^★★^
Normal	212	57.5	108	56.0	104	59.1		
Overweight	105	28.5	60	31.1	45	25.6		
Obese	8	2.2	5	2.6	3	1.7		

*Calculated BMI*								
Underweight	16	4.3	13	6.7	3	1.7		
Normal	234	63.4	113	58.5	121	68.8	8.209	0.042^*∗*^
Overweight	102	27.6	56	29.0	46	26.1		
Obese	17	4.6	11	5.7	6	3.4		

*Agreement between perceived and measured weight status*								
Agree	247	66.9	132	68.4	115	65.3	0.388	0.534
Did not agree	122	33.1	61	31.6	61	34.7		

*Perceived diet quality*								
Adequate	227	61.7	119	61.7	108	61.7	0.3571	0.903 (0.742)^★★^
Too much sugar	60	16.3	33	17.1	27	15.4		
Too much fat	55	14.9	29	15.0	26	14.9		
Not enough	26	7.1	12	6.2	14	8.0		

*Total calculated food score*	68.23 ± 6.63		68.85 ± 6.31		68.53 ± 6.48		*t* = 0.917	0.360

*Agreement between perceived and calculated diet quality*								
Agree	204	55.3	108	56.0	96	54.5	0.074	0.785
Did not agree	165	44.7	85	44.0	80	45.5		

*Current physical activity level compared to preuniversity life*								
Decreased	97	26.3	62	32.1	35	19.9	15.407^*∗*^	<0.001^*∗*^ (<0.001)^★★^
Same	127	34.4	73	37.8	54	30.7		
Increased	145	39.3	58	30.1	87	49.4		

*Perceived current physical activity status*								
Low	120	32.5	66	34.2	54	30.7	0.760	0.684 (0.387)^★★^
Moderate	213	57.7	110	57.0	103	58.5		
High	36	9.8	17	8.8	19	10.8		

*PA level based on MET values*								
Low	152	41.2	79	40.9	73	41.5	1.556	0.459
Moderate	178	48.2	90	46.6	88	50.0		
High	39	10.6	24	12.4	15	8.5		

*Agreement between perceived and measured physical activity*								
Agree	198	53.7%	944	48.7%	104	59.1%	3.993^*∗*^	0.046^*∗*^
Did not agree	171	46.3%	99	51.3%	72	40.9%		

^*∗*^Statistically significant at *p* ≤ 0.05.

^★★^Values between brackets represent the *p* values that resulted by linear test.

**Table 4:** Mean weekly consumption of selected food items.

	Total (*n* = 369)	Junior (*n* = 193)	Senior (*n* = 176)	*p*
Fresh fruit	12.59 ± 10.0	13.28 ± 10.93	11.82 ± 8.84	0.512
Fresh fruit juice	5.27 ± 8.16	6.44 ± 9.81	3.98 ± 5.59	0.081
Raw veg.	9.21 ± 9.91	8.77 ± 9.81	9.69 ± 10.02	0.189
Cooked veg.	4.86 ± 9.14	4.33 ± 8.55	5.45 ± 9.73	0.292
Milk (whole)	2.21 ± 6.20	2.66 ± 7.86	1.72 ± 3.55	0.765
Milk (semiskimmed)	0.94 ± 4.40	0.98 ± 4.53	0.90 ± 4.26	0.901
Pulses	3.12 ± 7.37	3.54 ± 8.60	2.66 ± 5.71	0.506
Corn/sunflower oil	11.05 ± 10.22	10.52 ± 9.61	11.63 ± 10.84	0.570
Olive oil	12.06 ± 11.81	11.10 ± 11.87	13.11 ± 11.68	0.035^*∗*^
Yoghurt (whole)	4.54 ± 9.52	5.88 ± 12.11	3.08 ± 5.07	0.502
Yoghurt (low)	1.35 ± 6.03	1.97 ± 7.39	0.67 ± 3.97	0.024^*∗*^
Fried potato	5.54 ± 8.38	6.05 ± 9.10	4.98 ± 7.49	0.125
Sugar	13.01 ± 13.09	13.14 ± 13.82	12.87 ± 12.28	0.593
Soft drinks	5.72 ± 9.70	6.74 ± 11.20	4.61 ± 7.61	0.130
Butter	4.11 ± 7.25	3.92 ± 6.64	4.32 ± 7.88	0.726
Chocolate	9.84 ± 11.33	9.99 ± 11.64	9.68 ± 11.01	0.887
Cake	3.13 ± 8.01	3.86 ± 9.59	2.34 ± 5.75	0.065
Croissant	2.31 ± 6.57	3.08 ± 7.87	1.47 ± 4.62	0.001^*∗*^
Pizza	2.02 ± 7.24	2.87 ± 9.15	1.10 ± 4.10	0.001^*∗*^
Burgers	1.45 ± 5.83	1.85 ± 7.10	1.01 ± 3.97	0.069
White bread	10.82 ± 7.31	11.28 ± 7.92	10.32 ± 6.55	0.490
Rice and pasta	8.10 ± 11.34	8.89 ± 12.79	7.24 ± 9.47	0.890
Cooked and grilled fish	1.05 ± 3.28	1.22 ± 4.06	0.87 ± 2.11	0.345
Total diet score				
Min.–max.	44.0–92.0	44.0–92.0	48.0–90.0	0.360
Mean ± SD	68.53 ± 6.48	68.23 ± 6.54	68.85 ± 6.31	
Median	69.0	68.0	69.0	

^*∗*^Statistically significant at *p* ≤ 0.05.

**Table 5:** Association between overweight/obesity and sociodemographic, dietary, and lifestyle behaviors among university students.

	BMI				
	*B*	Sig.	OR	95% CI	
				LL	UL
*Obesity of one parent*					
No®					
Yes	0.758	0.006^*∗*^	0.468	0.274	0.802

*Do you drink coffee or tea directly after meals?*					
No®					
Yes	−0.478	0.063	0.620	0.375	1.026

*Level*					
Senior®					
Junior	0.813	0.106	2.255	0.842	6.037

*Number of snacks*					
Zero®					
One	−0.133	0.811	0.876	0.296	2.591
Two	−0.326	0.550	0.722	0.248	2.100
Three	−1.150	0.057	0.317	0.097	1.036
More than three	−0.048	0.938	0.953	0.281	3.236

*Current appetite status compared to preuniversity life*					
Same®					
Increased	0.764	0.013^*∗*^	0.466	0.255	0.851
Decreased	−0.427	0.160	0.652	0.359	1.184

*Cravings for high fat/sugar foods*					
Never®					
Once/month	0.622	0.138	1.863	0.819	4.240
2–4 times/month	0.454	0.276	1.574	0.696	3.557
2-3 times/week	1.116	0.012^*∗*^	3.054	1.282	7.272
4 times/week	0.672	0.219	1.958	0.671	5.713

*Exams related stressful eating*					
No®					
Yes	0.397	0.151	1.488	0.865	2.560
Sometimes	0.983	0.016^*∗*^	2.672	1.198	5.960
Rarely	0.232	0.812	1.261	0.187	8.502

*Comfort eating*					
No®					
Yes	0.542	0.044^*∗*^	0.581	0.343	0.984

*Regular breakfast intake*					
Yes®					
No	0.246	0.347	1.279	0.766	2.134

*PA level based on MET values*					
Low	0.182	0.681	1.199	0.504	2.855
Moderate	0.095	0.827	1.099	0.470	2.572
High®					

*Total food score*	−0.053	0.010^*∗*^	1.055	1.013	1.099

^*∗*^Statistically significant at *p* ≤ 0.05.

®Reference.

## Additional Points

The cross-sectional study design in the present study does not allow disclosing trends of change in adiposity status and energy balance related behaviors in the same students. Secondly, data collection was through an interview (face to face) and not self-administered questionnaires, thus enabling researchers to explain the questionnaire in full prior to completion which could minimize the errors of self-reported data; yet, there is no gold standard method to overcome the recall bias.
